# Mechanisims of asthma and allergic disease – 1076. Identification of genes related to seasonal allergic rhinitis by microarray analysis

**DOI:** 10.1186/1939-4551-6-S1-P73

**Published:** 2013-04-23

**Authors:** Yoshimasa Imoto, Masafumi Sakashita, Takahiro Tokunaga, Takechiyo Yamada, Shigeharu Fujieda

**Affiliations:** 1 The Department of Otorhinolaryngology Head & Neck Surgery, University of Fukui, Fukui, Japan

## Background

The number of seasonal allergic rhinitis by Japanese cedar pollen (SAR-JCP) patients has been increasing in this decade in Japan. To identify genes that related to SAR-JCP, we analyzed gene expression patterns.

## Methods

We collected samples from February to April, at the time of JCP dispersion. Subjects were SAR-JCP patients (SAR-JCP group), subjects who had positive specific IgE against JC pollen but no symptoms (Sensitization group) and controls without any allergic symptoms and no elevated IgE to common environmental allergens (control group). Total RNA was extracted from nasal epithelial cells by brushing inferior turbinate and performed microarray analysis with Illumina Human Ref8 BeadChip arrays.

## Results

Microarray analysis revealed that the expressions of 16 genes were significantly altered in nasal epithelial cells during allergen exposure. Among these 16 genes, four genes including *ITLN1*(intelectin1) were significantly up-regulated during pollen seasons. Experiment using human nasal epithelial cells revealed that intelectin1 expression was induced by IL-4 and IL-13 stimulation.

## Conclusions

The present study identified alteration of genes during natural allergen exposure in patients with SAR-JCP, which may further elucidate the molecular mechanisms underlying SAR.

